# MAFG-AS1 is a prognostic biomarker and facilitates prostate cancer progression

**DOI:** 10.3389/fonc.2022.856580

**Published:** 2022-08-05

**Authors:** Peizhang Li, Yuanping Shi, Miaomiao Guo, Huan Xu, Ming Zhan, Zhong Wang, Yanbo Chen

**Affiliations:** ^1^ Department of Urology, Shanghai Ninth People’s Hospital Affiliated to Shanghai Jiaotong University School of Medicine, Shanghai, China; ^2^ Department of Endocrinology and Metabolism, Peking University People's Hospital, Beijing, China; ^3^ The Core Laboratory in Medical Center of Clinical Research, Department of Molecular Diagnostics and Endocrinology, Shanghai Ninth People’s Hospital, Shanghai Jiaotong University School of Medicine, Shanghai, China; ^4^ Department of Molecular Diagnostics, Shanghai Ninth People’s Hospital Affiliated to Shanghai Jiaotong University School of Medicine, Shanghai, China

**Keywords:** MAFG-AS1, lncRNAs, prostate cancer, prognosis, tumor-microarray

## Abstract

Long Noncoding RNAs (LncRNAs) have recently been identified as key regulator in tumor progression. The LncRNA MAFG-AS1 has been reported to facilitate the progression of multiple cancers, however, its role in prostate cancer is still unknown. Here, we reported that MAFG-AS1 was upregulated in prostate cancer. Importantly, high expression of MAFG-AS1 indicated advanced stage prostate cancer. Univariate and Multivariate Cox regression analyses showed that high MAFG-AS1 expression was independently correlated with poor progression-free interval (PFI). According to the result of The Cancer Genome Atlas (TCGA) database and tissue microarray, high MAFG-AS1 expression indicated a poor prognosis in prostate cancer patients. In addition, gene functional enrichment analysis revealed that MAFG-AS1 may be involved in ribosome biogenesis, ribonucleoprotein complex subunit organization, ribonucleoprotein complex assembly, rRNA metabolic process, structural constituent of ribosome, and ribonucleoprotein complex binding. Furthermore, MAFG-AS1 knockdown by siRNA markedly impaired prostate cancer cell proliferation, migration, and invasion.

## Introduction

Prostate cancer is one of the most common cancers in men worldwide. With regard to the cancer-related mortality of prostate cancer, it is currently ranked first in the US (1–3). According to the American Cancer Society (ACS), 161,360 new cases of prostate cancer accounted for the first incidence of malignant tumors in men (19%), and 26,730 new deaths from prostate cancer accounted for the third highest mortality rate among male malignant tumors (8%) in 2017 (4). Androgen deprivation therapy (ADT) is the first-line therapy for prostate cancer patients, and it has been proven to improve the overall survival (OS) of men diagnosed with metastatic prostate cancer (5). However, the tumor will subsequently progress to resistance to ADT and inevitably develop into castration-resistant prostate cancer (CRPC). Metastasis has become a bottleneck restricting the long-term survival of prostate cancer patients and is also the key to overcoming prostate cancer. The molecular mechanisms underlying the progression of prostate cancer remain largely unknown. Therefore, there is an urgent need to elucidate the underlying mechanisms of prostate cancer and explore novel molecular targets that are crucial for the development of new diagnostic and therapeutic drugs for the treatment of prostate cancer.

Long non coding RNAs (lncRNAs) are a large class of no protein-coding capacity transcripts that are longer than 200 nucleotides (6). lncRNAs have been reported to be involved in different biological processes, such as cell proliferation, apoptosis, angiogenesis, migration, invasion, and drug resistance (7–11). Accumulating evidence has demonstrated that dysregulation of lncRNAs is strongly associated with the development and progression of cancer. Furthermore, many lncRNAs have been reported to regulate the pathogenesis of prostate cancer (12). Previous studies have suggested that lncRNA NEAT1-1 is involved in bone metastasis of prostate cancer and promotes the binding ability between CYCLINL1 and CDK19 in an N6-methyladenosine dependent manner (13). Luo et al. showed that lncRNA-p21 is upregulated in neuroendocrine prostate cancer (NEPC) and overexpressed lncRNA-p21 induces the neuroendocrine differentiation (NED) (14). Mechanistically, lncRNA-p21 can disrupt the PRC2 complex and promote the methylation of STAT3 to induce NED. Several studies have demonstrated that MALAT1 upregulation promotes prostate cancer cell growth, migration, and invasion (15). Furthermore, the lncRNA MAFG-AS1 was upregulated in breast cancer and facilitated breast carcinoma progression by regulating MMP15 expression (16). MAFG-AS1 also promoted cell proliferation, migration, and invasion of hepatocellular cancer *via* targeting miR-3196/OTX1 axis (17). Additionally, MAFG-AS1 regulated tumorigenesis of colorectal cancer by acting as a sponge of miR-149-3p (18). However, the role of MAFG-AS1 in prostate cancer progression remains largely unknown.

In the current study, we investigated the expression of MAFG-AS1 in prostate cancer according to the TCGA database and a tissue microarray, and determined its role as a prognostic biomarker in prostate cancer. In addition, we searched for the gene set most related to the expression of MAFG-AS1, then predicted the functions and pathways of MAFG-AS1 in prostate cancer through gene enrichment analysis. Furthermore, to investigate the role of MAFG-AS1 in cell proliferation, migration, and invasion in prostate cancer, we performed a series of *in vitro* experiments.

## Methods and materials

### RNA-sequencing data and bioinformatics analysis

A total of 495 cases containing both gene expression data (HTSeq-Counts) and clinical information from the PRAD database were obtained from The Cancer Genome Atlas (TCGA) for further analysis. HTSeq-Counts data were transformed into transcripts per million reads (TPM). Data of 495 cases were used for survival analysis. Next, the characteristics of patients consists of T stage, N stage, Gleason score of pathologic of surgical specimens, and progression-free interval (PFI) result. Pathological T and N stage were performed according to the extent of tumor invasion and the presence of lymphatic metastasis. This study satisfied the publication requirements stated by TCGA (http://cancergenome.nih.gov/publications-/publicationguidelines).

### Cell lines and culture

The human prostate cancer cell line PC-3 originated from bone marrow metastases in a 62-year-old white male patient diagnosed with grade IV prostate cancer. Prostate cancer cells DU145 were established from a brain metastasis of a 69-year-old Caucasian patient with prostate cancer. The cells were obtained from the National Collection of Authenticated Cell Culture at the Chinese Academy of Science (Shanghai, China). PC-3 and DU145 cells were cultured in MEM supplemented with 10% fetal bovine serum (FBS) (Gibco, USA). Cell authentication was validated using STR profiling.

### Small interfere RNA (siRNA) construction

Small interfering RNAs (siRNAs) targeting MAFG-AS1 were obtained from GenePharma (Shanghai, China), and the sequence information targeting MAFG-AS1 was as follows: siMAFG-AS1-1, GGAGTCAGGGCAATTCCAA; siMAFG-AS1-2, GGTAACATAGAGACCCTAT.

### Total RNA isolation and real-time qPCR

Total RNA was extracted from prostate cancer cells using TRIzol reagent (Invitrogen, USA) according to the manufacturer’s protocol. Total RNA was then reverse transcribed to cDNA using random primers using a Revert Aid First Strand cDNA Synthesis kit (Thermo Fisher, USA). RT-qPCR was performed using TB Green Premix Ex Taq (Takara, Germany). GAPDH was used as an internal control. qPCR primers were synthesized from BioSune (Shanghai, China). The primers used were as follows: MAFG-AS1-F: CGGGAGGAAGATAAACGGGG, MAFG-AS1-R: TGACCACGGAACACCTTCAG, GAPDH-F: CTGGGCTACACTGAGCACC, GAPFH-R: AAGTGGTCGTTGAGGGCAATG.

### Cell proliferation assay and colony formation assay

For the cell proliferation assay, a total of 3000 prostate cancer cells were seeded into 96-well plates and incubated in 10% CCK-8 medium for one hour at 0 hour, 24 hours, 48 hours, and 72 hours after seeding. The absorbance was measured at 450 nm with a spectrophotometer. For the colony formation assay, prostate cancer cells were seeded in 6-well plates at a density of 200 cells per well and cultured for 2 weeks. The cells were then fixed and subsequently examined by crystal violet staining.

### Transwell migration assays and transwell invasion assay

For the transwell migration assay, 20000 cells were suspended in 200 μl of medium without FBS and were seeded in the upper chamber of transwell inserts (Corning, USA). 500 μl medium with 20% FBS were added into the lower chamber. The cells were then incubated for 18 h. For the transwell invasion assay, 30000 cells were suspended in 200 μl of medium without FBS and were seeded on the upper chamber of transwell inserts coated with Matrigel (BD Biosciences, USA). The cells were then cultured for 24 h. Cells were fixed with 4% PFA and stained with a crystal violet staining solution. Images were captured with a ×20 objective using a Leica DM LB light microscope and the number of cells was counted using ImageJ.

### Statistical analysis

All statistical analyses were conducted using SPSS (22.0). Pearson’s χ2 test was used to determine the correlation between MAFG-AS1 expression and clinicopathological variables. The t-test was used to determine statistically significant differences between the two groups. Kaplan–Meier analysis was performed to compare the survival time differences between the MAFG-AS1 high expression group and low expression group. The log-rank test p < 0.05 suggested the significance of survival time differences. The hazard risk of the individual indicators was estimated using hazard ratios (HRs) with 95% confidence intervals (CIs). All reported P-values were two-sided and P-values of less than 0.05 were considered to be significant. * represents P < 0.05, ** represents P < 0.01, and *** represents P < 0.001.

## Result

### MAFG-AS1 was highly expressed in prostate cancer

**Figure 1:**
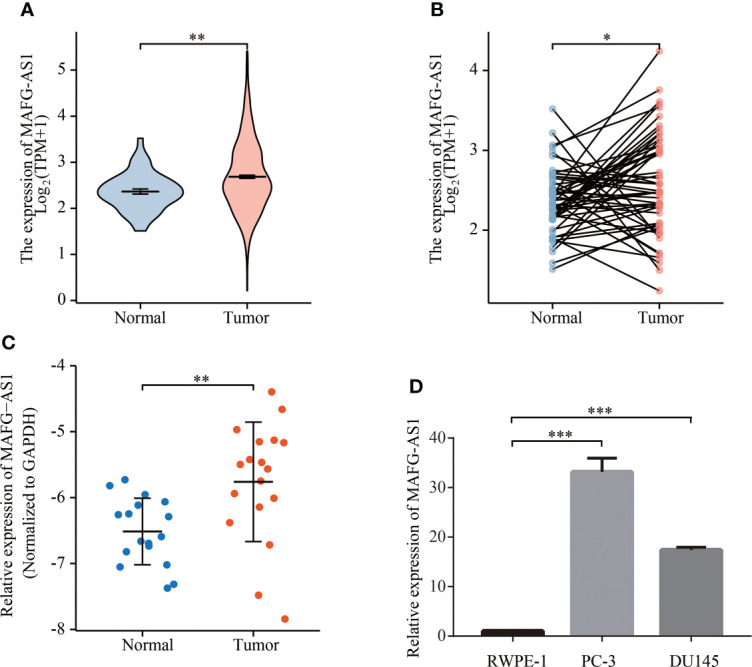
MAFG-AS1 was highly expressed in prostate cancer. **(A)** MAFG-AS1 expression in prostate cancer and adjacent normal tissue in TCGA database. **(B)** MAFG-AS1 expression in 50 pairs of prostate cancer and adjacent normal tissue in TCGA database. **(C)** MAFG-AS1 expression in prostate cancer and normal prostate tissue using RT-qPCR. **(D)** MAFG-AS1 expression in prostate cancer cell lines (PC-3, DU145) and normal prostate epithelial cell line (RWPE-1). Data were indicated as mean ± standard deviation, ns P ≥ 0.05, * P < 0.05, ** P < 0.01, *** P < 0.001..

The data collected from TCGA in October 2019 contained 495 tumor samples with both clinical information and gene expression data (**Table 1**) and the clinical features of the patients included age, TNM stage, Gleason scores, and PFI events. To evaluate the expression of MAFG-AS1 in prostate cancer, we compared MAFG-AS1 expression in 495 prostate cancer and 50 adjacent normal tissues, and the results suggested that MAFG-AS1 was upregulated in prostate cancer (**Figure 1A**). Similarly, by comparing 50 pairs of prostate cancer tissues and adjacent normal tissues, we also found that MAFG-AS1 was highly expressed in prostate cancer (**Figure 1B**). In addition, we collected 18 pairs of prostate cancer tissues and adjacent normal tissues, and the results of RT-qPCR demonstrated that MAFG-AS1 expression was upregulated in prostate cancer (**Figure 1C**). Next, the results of RT-qPCR indicated that MAFG-AS1 expression in prostate cancer cell lines PC-3 and DU145 was higher than that in the normal prostate epithelial cell line RWPE-1 (**Figure 1D**). These results demonstrated that MAFG-AS1 expression is overexpressed in prostate cancer.

**Table 1:** Correlation between MAFG-AS1 expression and clinicopathological characteristics of prostate cancer.

Characteristic	Low expression of MAFG-AS1	High expression of MAFG-AS1	p
n	249	250	
T stage, n (%)			<0.001
T2	113 (23%)	76 (15.4%)	
T3	128 (26%)	164 (33.3%)	
T4	3 (0.6%)	8 (1.6%)	
N stage, n (%)			0.010
N0	177 (41.5%)	170 (39.9%)	
N1	27 (6.3%)	52 (12.2%)	
M stage, n (%)			1.000
M0	224 (48.9%)	231 (50.4%)	
M1	1 (0.2%)	2 (0.4%)	
Age, n (%)			0.226
<=60	119 (23.8%)	105 (21%)	
>60	130 (26.1%)	145 (29.1%)	
PSA(ng/ml), n (%)			0.923
<4	212 (48%)	203 (45.9%)	
>=4	13 (2.9%)	14 (3.2%)	
Gleason score, n (%)			<0.001
6	34 (6.8%)	12 (2.4%)	
7	134 (26.9%)	113 (22.6%)	
8	30 (6%)	34 (6.8%)	
9	50 (10%)	88 (17.6%)	
10	1 (0.2%)	3 (0.6%)	
PFI event, n (%)			0.002
Alive	216 (43.3%)	189 (37.9%)	
Dead	33 (6.6%)	61 (12.2%)	
Primary therapy outcome, n (%)			0.012
PD	13 (3%)	15 (3.4%)	
SD	9 (2.1%)	20 (4.6%)	
PR	14 (3.2%)	26 (5.9%)	
CR	187 (42.7%)	154 (35.2%)	
Age, meidan (IQR)	61 (56, 66)	62 (57, 66)	0.231

### MAFG-AS1 was upregulated in advanced prostate cancer and indicated a poor prognosis in TCGA database

**Figure 2:**
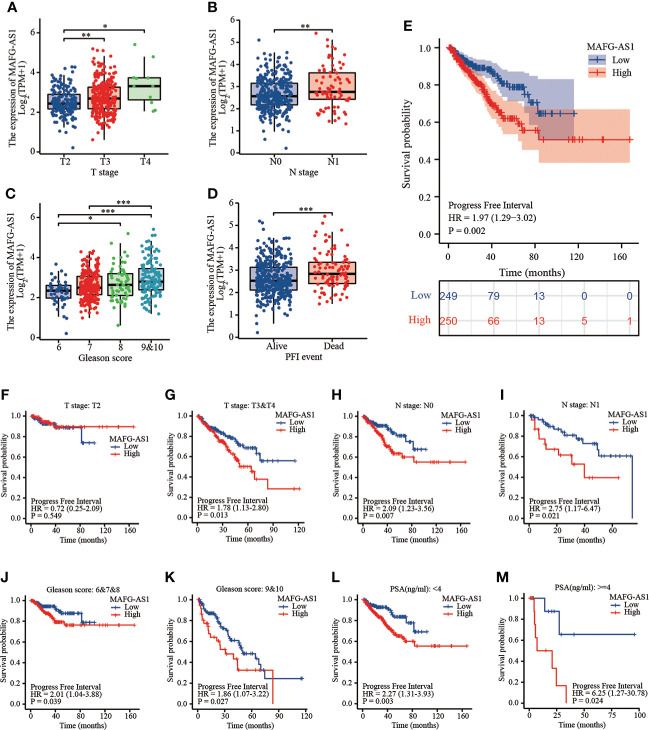
MAFG-AS1 was upregulated in advanced prostate cancer and indicated a poor prognosis. **(A)** MAFG-AS1 expression in prostate cancer with T3&T4 stage or T2 stage. **(B)** MAFG-AS1 expression in prostate cancer with N1 stage or N0 stage. **(C)** MAFG-AS1 expression in prostate cancer with Gleason score 6&7&8 or 9&10. **(D)** MAFG-AS1 expression in prostate cancer patients with different PFI. **(E)** Kaplan-Meier survival analysis revealed that prostate cancer patients with high MAFG-AS1 expression exhibited a shorter PFI. **(F, G)** Kaplan-Meier survival analysis of prostate cancer with different MAFG-AS1 level with T2 or T3&T4 stage. **(H, I)** Kaplan-Meier survival analysis of prostate cancer with different MAFG-AS1 level with N0 or N1 stage. **(J, K)** Kaplan-Meier survival analysis of prostate cancer with different MAFG-AS1 level with Gleason score 6&7&8 or 9&10. **(L, M)** Kaplan-Meier survival analysis of prostate cancer with different MAFG-AS1 level with PSA level < 4ng/ml or > 4ng/ml. Data were indicated as mean ± standard deviation, ns P ≥ 0.05, * P < 0.05, ** P < 0.01, *** P < 0.001..

Logistic regression analysis demonstrated that higher MAFG-AS1 expression, regarded as an independent variable, was correlated with better prognostic characteristics (**Table 2**). High MAFG-AS1 expression in the PRAD cohort was significantly associated with T classification (OR = 1.952 for T3&T4 *vs*. T2, P < 0.001), N classification (OR = 2.005 for N1 *vs*. N0, P = 0.008), and Gleason score (OR = 2.074 for 8&9&10 *vs*. 6&7, P < 0.001). These results revealed that prostate cancer with high MAFG-AS1 expression is more likely to be in a primitive stage than those with low MAFG-AS1 expression. The expression level of MAFG-AS1 was higher in the T3&T4 stage than in the T2 stage (**Figure 2A**). Similarly, MAFG-AS1 expression was higher in advanced prostate cancer according to N stage, Gleason scores, and PFI events (**Figures 2B–D**). Consistently, MAFG-AS1 high expression was significantly correlated with poor prognosis in patients with prostate cancer patients (**Figure 2E**). Furthermore, univariate Cox regression analysis showed that high MAFG-AS1 expression was correlated with a poor PFI (hazard ratio [HR]: 1.985; 95% confidence interval [CI]: 1.299- 3.035; P < 0.01), and other clinical variables, including advanced T stage, N stage, and Gleason score, remained associated with a poor prognosis (**Table 3**). Multivariate analysis suggested that high MAFG-AS1 expression was independently associated with poor PFI (HR = 1.78; CI: 1.121- 2.847; P = 0.015).

**Table 2:** MAFG-AS1 expression associated with clinical pathological characteristics (logistic regression).

Characteristics	Total (N)	Odds Ratio (OR)	P value
T stage (T3&T4 *vs*. T2)	492	1.952 (1.352-2.831)	<0.001
N stage (N1 *vs*. N0)	426	2.005 (1.213-3.378)	0.008
M stage (M1 *vs*. M0)	458	1.939 (0.185-41.906)	0.590
Gleason score (8&9&10 *vs*. 6&7)	499	2.074 (1.445-2.989)	<0.001
PSA (ng/ml) (>=4 *vs*. <4)	442	1.125 (0.514-2.480)	0.768

**Table 3:** Univariate and multivariate analysis of factors associated with PFI using Cox regression.

Characteristics	Total (N)	Univariate analysis		Multivariate analysis	
		Hazard ratio (95% CI)	P value	Hazard ratio (95% CI)	P value
T stage (T3&T4 *vs.* T2)	492	3.785 (2.140-6.693)	<0.001	3.386 (1.752-6.544)	<0.001
N stage (N1 *vs.* N0)	426	1.946 (1.202-3.150)	0.007	1.225 (0.732-2.051)	0.441
M stage (M1 *vs.* M0)	458	3.566 (0.494-25.753)	0.208		
PSA (ng/ml)	442	4.196 (2.095-8.405)	<0.001	2.616 (1.186-5.768)	0.017
MAFG-AS1 (High *vs.* Low)	495	1.985 (1.299-3.035)	0.002	1.787 (1.121-2.847)	0.015

We then performed a stratified analysis based on the clinical information of prostate cancer patients. KM-plot analysis revealed that high MAFG-AS1 expression was associated with a poor prognosis in T3&T4 stage patients with prostate cancer; however, MAFG-AS1 expression was not associated with prognosis in T2 stage patients (**Figures 2F, G**). In patients with or without lymph node metastasis, MAFG-AS1 expression could be used as a prognostic marker, indicating poor PFI (**Figures 2H, I**). Similarly, in patients with high or low Gleason scores, high expression of MAFG-AS1 was correlated with poor prognosis (**Figures 2J, K**). In addition, high MAFG-AS1 expression was associated with a poor prognosis in prostate cancer patients with PSA levels more than 4 ng/ml or less than 4 ng/ml (**Figures 2L, M**).

### MAFG-AS1 indicated a poor prognosis in tissue-microarray

**Figure 3:**
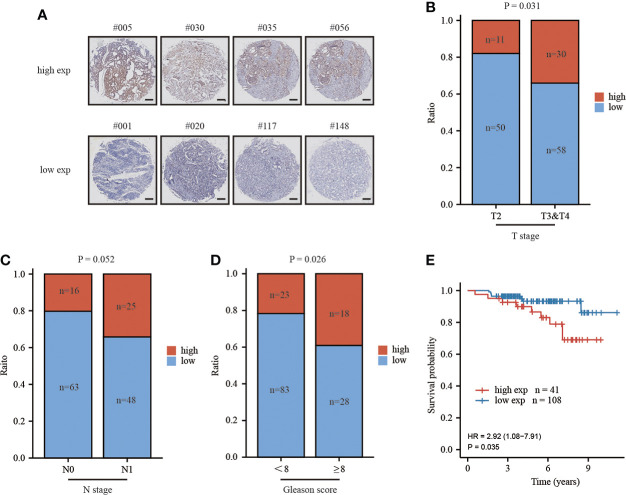
high expression of MAFG-AS1 indicated a poor prognosis. **(A)** Representative ISH results of prostate cancer patients in different groups (Scale bar: 200μm) **(B)** MAFG-AS1 expression in prostate cancer in T3&T4 or T2 stage by *in situ* hybridization (ISH). **(C)** MAFG-AS1 expression in prostate cancer in N1 or N0 stage by *in situ* hybridization (ISH). **(D)** MAFG-AS1 expression in prostate cancer in Gleason score<8 or ≥8. **(E)** High expression of MAFG-AS1 indicated a poor overall survival (OS) by Kaplan-Meier analysis.

To critically evaluate the prognostic value of MAFG-AS1 in prostate cancer, we performed a tissue microarray (**Figure 3A**; **Figure S1**), and the clinical information of TMA patient cohort was shown in **Table 4**. The ISH results demonstrated that MAFG-AS1 expression was higher in the T3&T4 stages than in the T2 stage (**Figure 3B**). Similarly, MAFG-AS1 was upregulated in the N1 stage compared to the N0 stage (**Figure 3C**). Furthermore, prostate cancer with a higher Gleason score showed higher MAFG-AS1 expression (**Figure 3D**). KM-plot analysis suggested that high expression of MAFG-AS1 indicated a poor overall survival (OS) in the TMA patient cohort (**Figure 3E**). These results demonstrate that MAFG-AS1 is correlated with prostate cancer clinical features, and high expression of MAFG-AS1 is associated with a poor prognosis in prostate cancer.

**Table 4:** Clinicopathological characteristics of prostate cancer patients in TMA cohort.

Characteristic	high exp	low exp	p
n	41	108	
T stage, n (%)			0.031
T2	11 (7.4%)	50 (33.6%)	
T3&T4	30 (20.1%)	58 (38.9%)	
N stage, n (%)			0.052
N0	16 (10.7%)	63 (42.3%)	
N1	25 (16.8%)	45 (30.2%)	
Gleasion scores, n (%)			0.026
<8	23 (15.4%)	83 (55.7%)	
≥8	18 (12.1%)	25 (16.8%)	
ages (years), mean ± SD	66.98 ± 7.16	68.92 ± 5.89	0.093

### Functional enrichment analysis of MAFG-AS1 in prostate cancer

**Figure 4:**
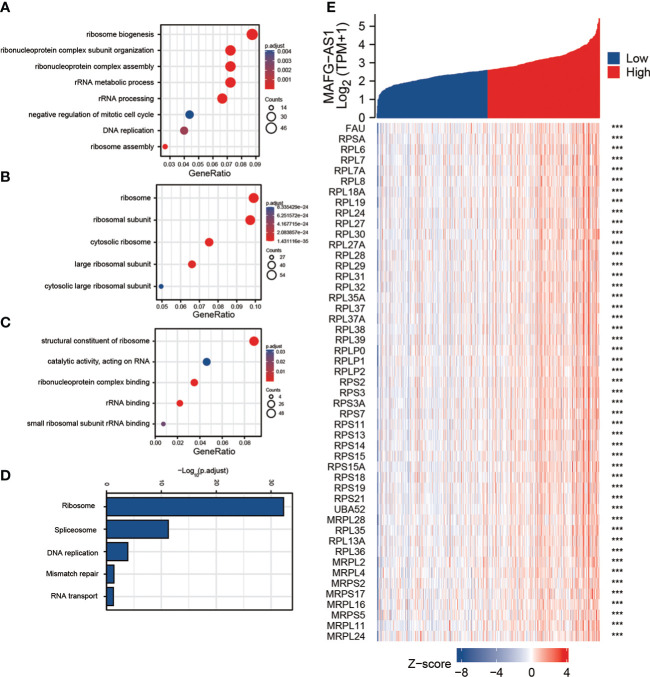
Gene functional enrichment analysis of the most relative genes with MAFG-AS1 expression. **(A)** Gene Ontology analysis of MAFG-AS1 related genes in biological process (BP). **(B)** Gene Ontology analysis of MAFG-AS1 related genes in cellular component (CC). **(C)** Gene Ontology analysis of MAFG-AS1 related genes in molecular function (MF). **(D)** Kyoto Encyclopedia of Genes and Genomes (KEGG) pathway analysis of MAFG-AS1 related genes. **(E)** The correlation analysis between MAFG-AS1 and ribosome related genes. ns P ≥ 0.05, * P < 0.05, ** P < 0.01, *** P < 0.001.

Next, we performed a functional gene enrichment analysis of MAFG-AS1. We searched for the top 500 genes related to MAFG-AS1 expression according to the TCGA-PRAD database. The results showed that these genes were enriched in ribosome biogenesis, ribonucleoprotein complex subunit organization, ribonucleoprotein complex assembly, and rRNA metabolic process in biological process (BP). Results also showed that these genes were enriched in ribosome and ribosomal subunit in cellular component (CC), and enriched in structural constituent of ribosome and ribonucleoprotein complex binding in molecular function (MF) (**Figures 4A–C**). Kyoto Encyclopedia of Genes and Genomes (KEGG) pathway analysis revealed that MAFG-AS1 related genes were enriched in the ribosome and DNA replication pathways (**Figure 4D**).

### The correlation analysis between MAFG-AS1 and ribosome related genes

Since functional enrichment analysis revealed that MAFG-AS1 may be involved in ribosome biogenesis, we investigated the correlation between MAFG-AS1 and ribosome-related genes. Among the top 500 genes most related to MAFG-AS1 expression, a number of genes were the components of ribosomes or involved in ribosome biosynthesis. For example, MAFG-AS1 expression was positively correlated with 60S ribosomal proteins (RPL6, RPL7, RPL7A, RPL8, etc.) and 40S ribosomal proteins (RPS2, RPS3, RPS3A, RPS7, etc.) (**Figure 4E**). Collectively, these results suggest that MAFG-AS1 may be involved in ribosome biogenesis to regulate prostate cancer tumorigenicity.

### MAFG-AS1 knockdown significantly impaired prostate cancer cell proliferation, migration and invasion

**Figure 5:**
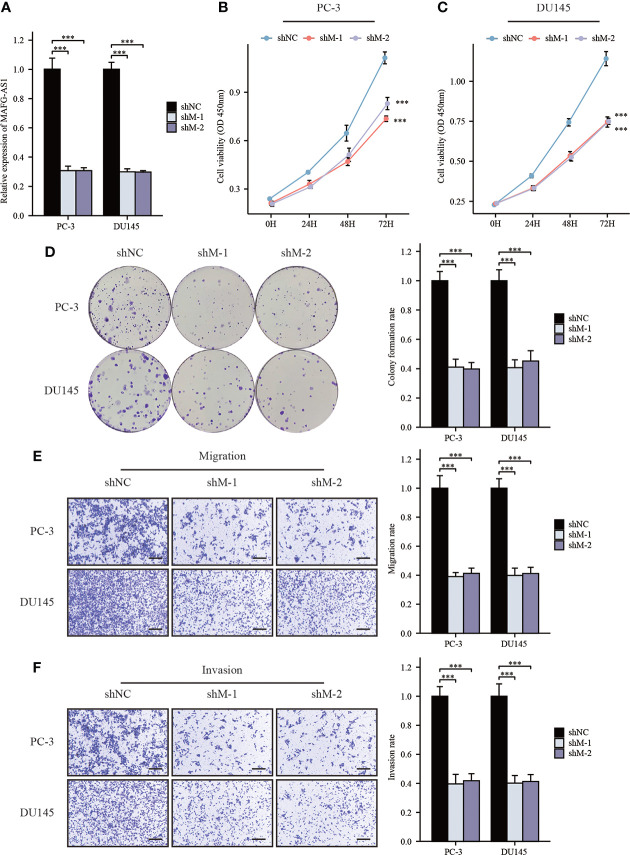
MAFG-AS1 knockdown significantly inhibited prostate cancer cell progression. **(A)** RT-qPCR analysis of MAFG-AS1 in MAFG-AS1 knockdown prostate cancer cells. **(B, C)** Downregulated MAFG-AS1 markedly inhibited cell viability of PC-3 and DU145 cells. **(D)** Knockdown of MAFG-AS1 impaired the ability of colony formation of PC-3 and DU145 cells. **(E, F)** The migration and invasion ability of PC-3 and DU145 cells were reduced following MAFG-AS1 knockdown (Scale bar: 50μm). Data were indicated as mean ± standard deviation, ns P ≥ 0.05, * P < 0.05, ** P < 0.01, *** P < 0.001.

To further elucidate the role of MAFG-AS1 expression in prostate cancer, we selected two prostate cancer cell lines (PC-3 and DU145) for subsequent research. RT-qPCR results showed that MAFG-AS1 expression was effectively downregulated in PC-3 and DU145 cells transfected with si-MAFG-AS1 (**Figure 5A**). The results of the CCK-8 assay demonstrated that MAFG-AS1 knockdown significantly inhibited prostate cancer cell viability (**Figures 5B, C**). The colony formation assay demonstrated that downregulation of MAFG-AS1 decreased the colony formation rate in prostate cancer cells (**Figure 5D**). For further studies, we conducted a transwell assay to clarify the role of MAFG-AS1 in prostate cancer migration and invasion. The results suggested that migration and invasion abilities were prominently impaired in MAFG-AS1 knockdown prostate cancer cells (**Figures 5E, F**). Taken together, we demonstrated that MAFG-AS1 knockdown significantly inhibited prostate cancer cell proliferation, migration, and invasion.

## Discussion

Several studies have highlighted the potential of MAFG-AS1 as a therapeutic target for cancer treatment. Mechanistically, MAFG-AS1 acts as a microRNA sponge to regulate tumorigenesis (16–20). For example, MAFG-AS1 facilitates esophageal squamous cell cancer progression by regulating miR143/LASP1 (19). MAFG-AS1 promotes the progression of pancreatic cancer by acting as a sponge for miR-3196 (20). In addition, MAFG-AS1 inhibited the stability of P53 to support cancer cell survival and division. Mechanistically, MAFG-AS1 binds to P53 and competitively inhibits TRIML2-mediated P53 SUMOylation and promotes the degradation of P53 by polyubiquitination (21). However, the role of MAFG-AS1 in prostate cancer has not been clearly elucidated.

Bioinformatics analysis showed that MAFG-AS1 expression was elevated in prostate cancer compared with normal prostate tissue and was higher in more advanced prostate cancer, indicating that MAFG-AS1 is a diagnostic biomarker for prostate cancer. In addition, KM-plot analysis and Cox regression analysis suggested that high expression of MAFG-AS1 was associated with a poor prognosis, and a series of functional experiments demonstrated that MAFG-AS1 knockdown significantly impaired prostate cancer cell progression. This indicates that MAFG-AS1 is a potential therapeutic target in prostate cancer.

Ribosomes are intracellular organelles that are responsible for translation of messenger RNAs (mRNAs) into functional proteins. Eukaryotes have 80S ribosomal subunits composed of large (60S) and small (60S) subunits. The 60S subunit consists of 5S rRNA, 5.8S rRNA, 28S rRNA, and approximately 47 proteins (RPL). The 40S subunit consists of 18S rRNA and approximately 33 proteins (RPS) (22, 23). Ribosomes play a pivotal role in the maintenance of cell growth, proliferation, and differentiation. Ribosomal dysfunction can lead to various diseases (24–26). Additionally, a large amount of evidence has demonstrated that ribosomal proteins are involved in tumor progression (27–30). In this study, we discovered that MAFG-AS1 expression was related to a number of RPLs and RPSs, and the genes most closely related to MAFG-AS1 expression were enriched in ribonucleoprotein complex subunit organization and ribonucleoprotein complex assembly, suggesting that MAFG-AS1 may be involved in ribosome biogenesis. However, further experimental evidence is needed to prove our hypothesis.

## Conclusion

In summary, MAFG-AS1 may play an important role in the occurrence and development of prostate cancer by regulating ribosome biogenesis. MAFG-AS1 may serve as a biomarker for the early diagnosis of prostate cancer and serve as a target for the treatment of prostate cancer.

## Data availability statement

The raw data supporting the conclusions of this article will be made available by the authors, without undue reservation.

## Ethics statement

The studies involving human participants were reviewed and approved by ethics committee of Shanghai Ninth People’s Hospital. The patients/participants provided their written informed consent to participate in this study.

## Author contributions

PL and YS conducted all the experiments. PL participated in the design of the study and drafted the manuscript. MG, HX, and MZ conducted the statistical analysis. YC and ZW designed the project and finalized the manuscript. All authors contributed to the article and approved the submitted version.

## Funding

This study was supported by Scientific research project of Shanghai Municipal Health Commission (20214Y0408), multi-center clinical research project of Shanghai JiaoTong University School of Medicine (DLY201809), and Shanghai Huangpu District Industry Support Fund (XK2020011), and Technology Transfer Project of Shanghai Jiao Tong University School of Medicine (ZT202110).

## Conflict of interest

The authors declare that the research was conducted in the absence of any commercial or financial relationships that could be construed as a potential conflict of interest.

## Publisher’s note

All claims expressed in this article are solely those of the authors and do not necessarily represent those of their affiliated organizations, or those of the publisher, the editors and the reviewers. Any product that may be evaluated in this article, or claim that may be made by its manufacturer, is not guaranteed or endorsed by the publisher.
