# Supplementary figures and images for: MAFG-AS1 is a prognostic biomarker and facilitates prostate cancer progression
Source: Front Oncol. 2022 Aug 5;12:856580. doi: 10.3389/fonc.2022.856580 (PMC9389335; doi:10.3389/fonc.2022.856580)

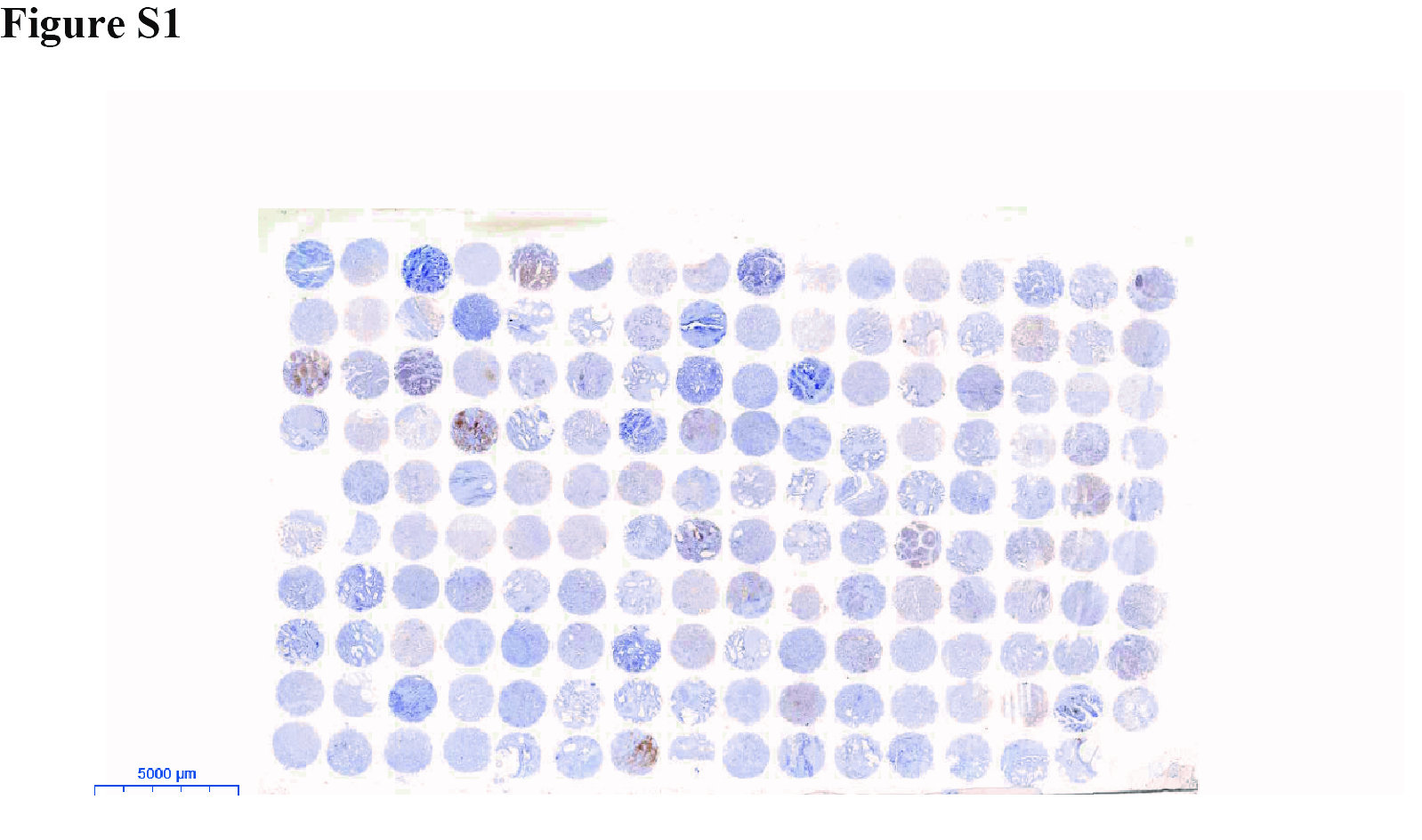

Supplement: Supplementary file 1 [file Image_1.tif]
